# Supplementary material for: Beneficial Effects of Dietary Fiber in Young Barley Leaf on Gut Microbiota and Immunity in Mice
Source: Molecules. 2024 Apr 22;29(8):1897. doi: 10.3390/molecules29081897 (PMC11054971; doi:10.3390/molecules29081897)
Supplement: Supplementary file 1 [file molecules-29-01897-s001.zip › molecules-2956450-supplementary.pdf]

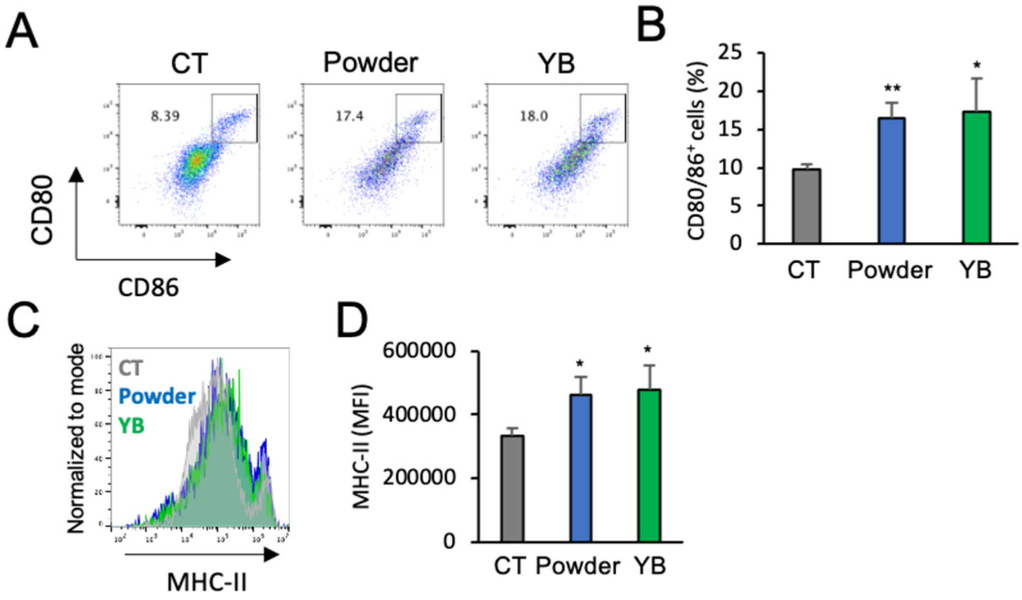

Supplementary Figure S1. Effect of young barley leaf powder (Powder, 5 mg/mL) or YB (3.7 mg/mL) on the BMDCs. (**A, B**) The percentage of CD80/86 positive cells in CD11c$^+$ BMDCs (n = 3). (**C, D**) The expression of MHC-II in CD11c$^+$ BMDCs (n = 4). the mean ± SD. Dunnett's multiple comparison test was used for the statistical analysis. *$p < 0.05$; **$p < 0.01$ versus CT.

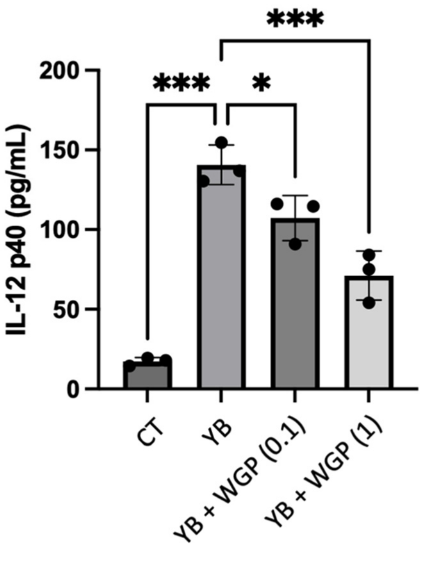

Supplementary Figure S2. Effect of Dectin-1 antagonist (WGP: 0.1 or 1 mg/mL) on IL-12 p40 production from BMDCs with YB treatment (n = 3). IL-12 p40 was measured by ELISA. Tukey's HSD post-hoc one-way ANOVA was performed for statistical analysis. *$p < 0.05$; *** $p < 0.001$.

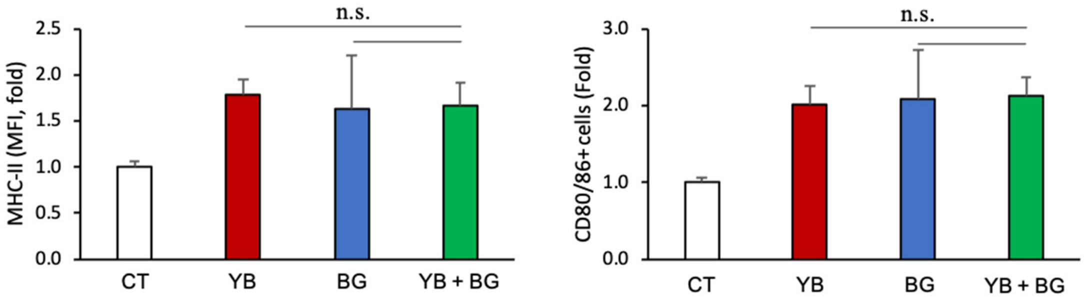

Supplementary Figure S3. Effect of YB and Dectin-1 agonist (b-glucan: BG, 10 mg/mL) on MHC-II and CD80/86 expression in BMDCs (n = 3). Tukey's HSD post-hoc one-way ANOVA was performed for statistical analysis. n.s.: not significant.

Supplementary Table S1. α-diversity and statistical values.

	CT	YB	H	*p*-value	*q*-value
shannon	4.626±0.209	4.939±0.189	0.833	0.361	0.361
obeseved_features	131.4±16.65	158.12±12.16	1.200	0.273	0.273
faith_pd	10.44±0.650	11.63±0.380	1.633	0.201	0.201
evenness	0.662±0.011	0.677±0.020	0.300	0.584	0.584
chao1	134.2±17.06	160.2±12.35	0.837	0.360	0.360

Supplementary Table S2. Statistical values at phylum level. *$q < 0.05$

Phylum	*p*-value	*q*-value
Firmicutes	0.518	0.865
Bacteroidota (Bacteroidetes)	0.972	0.972
Desulfobacterota	0.931	0.972
Deferribacterota	0.0329	0.098
Actinobacteriota	0.577	0.865
Proteobacteria	0.00728	0.043*
